# Effects of 17,18-Epoxyeicosatetraenoic Acid and 19,20-Epoxydocosapentaenoic Acid Combined with Soluble Epoxide Hydrolase Inhibitor *t*-TUCB on Brown Adipogenesis and Mitochondrial Respiration

**DOI:** 10.3390/nu17060936

**Published:** 2025-03-07

**Authors:** Yang Yang, Haoying Wu, Xinyun Xu, Christophe Morisseau, Kin Sing Stephen Lee, Bruce D. Hammock, Jiangang Chen, Ling Zhao

**Affiliations:** 1 Department of Nutrition, University of Tennessee, Knoxville, TN 37996, USA; yyang232@jhmi.edu (Y.Y.); hwu26@vols.utk.edu (H.W.); xxu28@vols.utk.edu (X.X.); 2 Department of Entomology and Nematology, Comprehensive Cancer Center, University of California, Davis, CA 95616, USA; chmorisseau@ucdavis.edu (C.M.); bdhammock@ucdavis.edu (B.D.H.); 3 Department of Pharmacology and Toxicology, Michigan State University, East Lansing, MI 48824, USA; sing@msu.edu; 4 Department of Chemistry, Michigan State University, East Lansing, MI 48824, USA; 5 Institute for Integrative Toxicology, Michigan State University, East Lansing, MI 48823, USA; 6 Department of Public Health, University of Tennessee, Knoxville, TN 37996, USA; jchen38@utk.edu

**Keywords:** 17,18-EEQ, 19,20-EDP, *n*-3 epoxy fatty acid, brown adipogenesis, thermogenesis, soluble epoxide hydrolase, soluble epoxide hydrolase inhibitor *t*-TUCB

## Abstract

**Background/Objectives**: 17,18-epoxyeicosatetraenoic acid (17,18-EEQ) and 19,20-epoxydocosapentaenoic acid (19,20-EDP) are bioactive metabolites produced from eicosapentaenoic acid (EPA) and docosahexaenoic acid (DHA), respectively, by CYP450s. These metabolites are unstable and quickly metabolized by auto-oxidation, esterification, β-oxidation, or hydrolysis by soluble epoxide hydrolase (sEH). 17,18-EEQ or 19,20-EDP combined with a potent sEH inhibitor *t*-TUCB differentially activated brown adipose tissue in diet-induced obesity. In the current study, we investigated whether these *n*-3 epoxy fatty acids with *t*-TUCB directly promote brown adipocyte differentiation and their thermogenic capacities. **Methods**: Murine brown preadipocytes were treated with 17,18-EEQ or 19,20-EDP with *t*-TUCB during and post differentiation. Brown marker protein expression and mitochondrial respiration were measured. In addition, the activation of PPARγ and suppression of NFκB reporter by 17,18-EEQ or 19,20-EDP alone or with *t*-TUCB were assessed, and the roles of PPARγ were evaluated with PPARγ knockdown and GW9662. **Results**: 17,18-EEQ or 19,20-EDP with *t*-TUCB promoted brown adipogenesis and mitochondrial respiration and uncoupling. Moreover, with *t*-TUCB, both epoxides improved mitochondrial respiration, but only 17,18-EEQ with *t*-TUCB significantly increased mitochondrial uncoupling (and heat production) in the differentiated adipocytes. PPARγ may be required for the effects of epoxides on differentiation but not on the thermogenic function post differentiation. **Conclusions**: The results demonstrate that, with *t*-TUCB, 17,18-EEQ and 19,20-EDP promote brown adipogenesis and mitochondrial respiration and uncoupling. 17,18-EEQ also promotes thermogenesis in differentiated brown adipocytes. Together, the results suggest thermogenic potentials of tested *n*-3 epoxides, especially 17,18-EEQ with *t*-TUCB. Translational studies of these *n*-3 epoxides on human brown adipocyte differentiation and functions are warranted.

## 1. Introduction

Obesity remains the most pressing chronic disease in the US and around the world [1,2]. Obesity increases the risks of developing cardiovascular diseases, diabetes, and some forms of cancer [3]. Lifestyle interventions with anti-obesity medications (AOM) are recommended for obese patients who are not candidates for surgical procedures [4]. Recently, glucagon-like peptide-1 receptor agonists (GLP1-RA), acting by reducing food intake, have shown weight loss benefits in clinical trials [5]; however, concerns about side effects, particularly in high-risk patients, from the long-term use of GLP-1RA have been raised [6]. No effective agents that increase energy expenditure have reached clinics.

*N*-3 epoxy fatty acids (EpFAs), such as epoxyeicosatetraenoic acids (EEQs) and epoxydocosapentaenoic acids (EDPs), are produced from eicosapentaenoic acid (EPA) and docosahexaenoic acid (DHA) by CYP450s, respectively [7,8]. 17,18-EEQ and 19,20-EDP are the predominant EEQ and EDP regioisomers found in vivo [8]. These *n*-3 EpFAs have been shown to modulate platelet aggregation [8], nociceptive signaling [9], thermogenesis, and cold tolerance [10]. However, they are unstable and can be quickly metabolized into less active diols by soluble epoxide hydrolase (sEH), a cytosolic enzyme encoded by the *Ephx2* gene and expressed in various tissues [8]. Therefore, potent pharmacological sEH inhibitors have been developed and are widely used to study the functional effects of EpFAs, and many of the beneficial effects of EpFAs were shown to be potentiated by the co-administration of an sEH inhibitor [8,11,12].

Dietary *n*-3 PUFA supplementation increased energy expenditure and thermogenesis in animals [13,14], and *n*-3 PUFAs, EPA in particular, also promoted brown adipogenesis and browning in vitro [15,16,17]. However, *n*-3 PUFAs were used at higher doses (e.g., 10–110 g/kg/day in rodent studies or up to 200 µM in mice-derived cells) (reviewed in [14]), and the effective metabolites were unknown. Recently, we demonstrated that 17,18-EEQ and 19,20-EDP (at 0.05 mg/kg/day) with an sEH inhibitor trans-4-{4-[3-(4-trifluoromethoxy-phenyl)-ureido]-cyclohexyloxy-benzoic acid (*t*-TUCB, 3 mg/kg/day) via osmotic pump delivery to the interscapular BAT (iBAT) effectively regulated protein expression in the iBAT and prevented the high-fat diet-induced metabolic dysfunction in mice [10]. As iBAT primarily consists of stromal precursor cells, which are capable of differentiation, and mature brown adipocytes, in the current study, we explored whether these EpFAs combined with *t*-TUCB have direct effects on brown adipocyte differentiation and thermogenic functions in differentiated brown adipocytes using a murine brown cell line.

## 2. Materials and Methods

### 2.1. Reagents

sEH inhibitor *t*-TUCB, 17,18-EEQ, and 19,20-EDP were synthesized as described in [18,19]. PPARγ receptor agonist rosiglitazone (Rosi) and antagonist GW9662 and bisphenol A diglycidyl ether (BADGE) were purchased from Cayman Chemical (Ann Arbor, MI, USA) and Sigma-Aldrich (St Louis, MO, USA), respectively. Anti-uncoupling protein 1 (UCP1) antibody (Catalog# U6382) was purchased from Sigma Aldrich (St. Louis, MO, USA) or from Cell Signaling Technology (Catalog# 72298) (Danvers, MA, USA). Anti-Peroxisome proliferator-activated receptor-gamma coactivator-1 alpha (PGC-1α) antibody (Catalog# AB3242) was purchased from Millipore (Temecula, CA, USA). Anti-CD36 (Catalog# NB400-144) was purchased from Novus Biologicals (Centennial, CO, USA).

### 2.2. Cell Culture and Treatment

A murine brown preadipocyte cell line was generated as described in [20]. The cells were maintained in growth media containing 20% fetal bovine serum (FBS) (Bio-Techne, Minneapolis, MN, USA) in Dulbecco’s Modified Eagle’s medium (DMEM) in an environment at 37 °C with 5% CO_2_ until they reached full confluence. The cells were then induced to differentiate (designated as day 0) in differentiation media containing DMEM supplemented with 20% FBS, 1 nM T_3_, and 20 nM insulin. The media was changed every 2 days for 6 days (day 6) to complete the differentiation program.

For experiments that studied the effects on brown preadipocyte differentiation (during differentiation), cells were treated with DMSO (the vehicle control, 0.1%), *t*-TUCB (T) alone, or with 17,18-EEQ (T+EEQ) or 19,20-EDP (T+EDP) at the indicated concentrations on day 0 (D0) of the differentiation program and replaced with each change in media until day 6 (D6). For experiments that studied the effects on mature brown adipocytes (post differentiation), cells were induced to differentiate in the differentiation media described above and then maintained in growth media in the presence of the DMSO, *t*-TUCB (T) alone, or with 17,18-EEQ (T+EEQ) or 19,20-EDP (T+EDP) at the indicated concentrations for 3 days.

To study the roles of PPARγ in the effects on brown differentiation by T alone or combined with epoxides, we employed murine brown preadipocyte cell line with PPAR knockdown and scrambled controls, as described in [21,22]. The treatment procedures were the same as the “during differentiation” protocol described above.

To study PPARγ antagonism in the effects on brown adipocytes post differentiation due to epoxides combined with T, cells were induced to differentiate, followed by maintenance in the DMEM supplemented with 20% FBS, 1 nM T_3_, and 20 nM insulin for 5 days. Then, the cells were pre-treated with GW9662 (20 µM) or the vehicle control (DMSO) for 2 h, followed by co-treatment with DMSO, T alone, T+EEQ, or T+EDP for 3 days, as the “post differentiation” protocol described above.

### 2.3. Oil Red O Staining

Lipid accumulation in the differentiated brown adipocytes was assessed by oil red O (ORO) staining and ORO absorbance, as described in [23].

### 2.4. Western Blot Analysis

Total cell lysates were prepared using RIPA lysis buffer (Cell Signaling, Danvers, MA, USA). Protein concentrations were determined by the BCA assay kit (Thermo Scientific, Waltham, MA, USA). Furthermore, 10% SDS-PAGE gels were used to separate protein samples. Proteins in the gels were then transferred to polyvinylidene difluoride membranes (Bio-Rad, Hercules, CA, USA) at 25 volts overnight. TBST buffer, containing 20 mM Tris Base, 137 mM NaCl, and 0.1% Tween 20 (pH 7.4)) supplemented with 5% nonfat milk, was used to block the membranes for 1 h. After sufficient washes, membranes were immunoblotted with the indicated primary antibodies against proteins of interest at 4 °C overnight at 1:1000 dilution. Next, membranes were washed thoroughly and incubated in secondary antibodies conjugated with horseradish peroxidase at 1:4000 dilution for 1 h. After thorough washes, the proteins of interest on the membranes were detected with SuperSignal West Pico Chemiluminescent Substrate (Thermo Scientific, Pittsburgh, PA, USA), and visualized and quantified by ChemiDoc XRS+ system with Image Lab 6.1 software (Bio-Rad, Hercules, CA, USA).

### 2.5. Analysis of Mitochondrial Respiration

For experiments using the “during differentiation” program, murine brown preadipocytes were differentiated in the presence or absence of DMSO, *t*-TUCB alone (TUCB, 1 μM), or with 17,18-EEQ (1 or 10 µM) (T+EEQ 1 or T+EEQ 10) or 19,20-EDP (1 or 10 µM) (T+EDP 1 or T+EDP 10) for 4 days.

For experiments using the “post differentiation” program, cells were induced to differentiate and then maintained in the growth media in the presence or absence of DMSO, *t*-TUCB (TUCB, 1 μM) alone, or with 17,18-EEQ (10 μM) (T+EEQ 10) or 19,20-EDP (10 μM) (T+EDP 10) for 3 days. To study the PPARγ antagonism, the cells were pre-treated with GW9662 or DMSO for 2 h, then co-treated with DMSO, T alone, T+EEQ, or T+EDP for 3 days, as described above.

Then, the cells were reseeded onto 24-well XFe assay plates at 2.0–5.0 × 10^4^cells per well. On the next day, cells were washed with XF assay media (XF assay base medium supplemented with 1 μM sodium pyruvate, 10 μM glucose, 2 μM glutamax, and 2% BSA) twice with a volume of 400 μL each. Then, the assay plate was added with 525 μL XF assay media into each well and incubated in a non-CO_2_ incubator (maintained at 37 °C) for 1 h. The real-time oxygen consumption rates (OCR) and extracellular acidification rates were measured at the basal level and then in response to the injections of oligomycin (1.5 µM), carbonyl cyanide-ptrifluoromethoxyphenylhydrazone (FCCP; 6 µM), and antimycin A/rotenone (R & A) (1 µM each) by an XFe24 Extracellular Flux Analyzer (Seahorse Biosciences, North Billerica, MA, USA). OCR and ECAR were automatically recorded by XFe24 software v1.8 provided by the manufacturer. Basal OCR, ATP production coupled OCR, proton leak coupled OCR, coupling efficiency, and maximal respiration capacity were calculated based on the manufacturer’s instructions.

### 2.6. Reporter Gene Assays

Brown preadipocytes were seeded and transiently transfected with murine peroxisome proliferator-activated receptor gamma (PPARγ) or NFκB activation reporters and β-galactosidase (β-gal) control plasmid with Lipofectamine 2000 transfection reagent and Plus reagent (Thermo Fisher Scientific, Carlsbad, CA, USA) for 24 h. PPARγ transactivation reporters consist of two separate systems: murine PPARγ ligand-binding domain ligated to the Gal4 DNA-binding domain (DBD) (mPPARγ-Gal4) and a reporter construct containing an upstream activating sequence (UAS)-linked luciferase, 4xUAS-TK-Luc (TK: thymidine kinase) [21,23]. NFκB activation reporter consists of an upstream NFκB-response element-linked luciferase, NFκB-Luc [24]. For PPARγ activation, the cells were treated with DMSO, 17,18-EEQ at 1 or 10 μM (EEQ 1 or EEQ 10) or 19,20-EDP at 1 or 10 μM (EDP 1 or EDP 10) alone, or with *t*-TUCB (1 μM) for 24 h. For NFκB activation, the cells were pre-treated with DMSO, 17,18-EEQ (10 µM), or 19,20-EDP (10 µM), alone or with *t*-TUCB (1 μM), for 1 h and then co-treated with ultra-pure lipopolysaccharide (LPS, 100 ng/mL) for 18 h. Cell lysates were prepared for reporter luciferase and β-gal activity measurements by GloMax Luminometer (Promega, Madison, WI, USA). Relative luciferase activity was normalized for β-gal activity and indicated as fold compared to the (-) group.

### 2.7. Statistical Analysis

Data are presented as Mean ± SEM, where n = 3 of biological replicates unless indicated otherwise for ORO absorbance, western blot analysis, and reporter assays and n = 3–6 for mitochondrial respiration assays on XFe 24 assay plates. Statistical analysis was performed using Prism 10 (GraphPad Software, San Diego, CA, USA). As indicated in figure legends, one-way or two-way ANOVA and unpaired *t*-tests were performed. The level of significance was set at *p* < 0.05.

## 3. Results

### 3.1. 17,18-EEQ and 19,20-EDP Combined with t-TUCB Differentially Promote Murine Brown Adipogenesis and Upregulate Protein Expression of Thermogenic and Lipid Metabolic Genes

**Figure 1:**
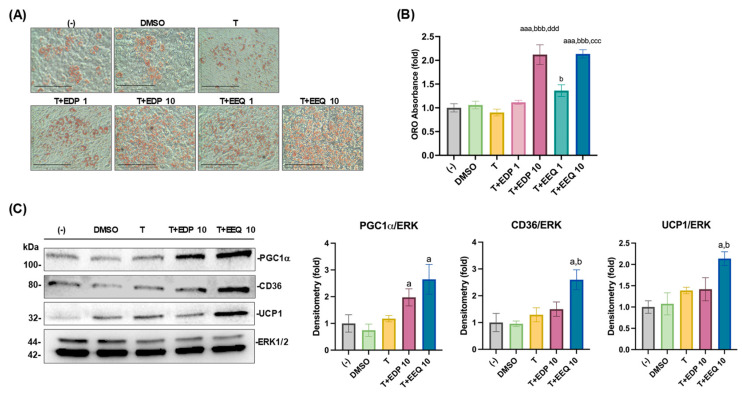
17,18-EEQ and 19,20-EDP combined with *t*-TUCB promoted murine brown adipogenesis. Murine brown preadipocytes were differentiated in the presence or absence of DMSO (the vehicle control), *t*-TUCB alone (T), or with 17,18-EEQ (T+EEQ) or 19,20-EDP (T+EDP) for 6 days. ORO-stained brown adipocyte morphology (**A**) and ORO absorbance (**B**) were shown. Protein expression of brown adipocyte marker genes, PGC1α, CD36, and UCP1 and the loading control ERK1/2 is shown in (**C**). Quantification of each protein expression by densitometry is shown on the right in (**C**). Data = Mean ± SEM (n = 3). One-way ANOVA was used in (**B**). a, *p* < 0.05, aaa, *p* < 0.001 compared to DMSO; b, *p* < 0.05, bbb, *p* < 0.001 compared to T; ccc, *p* < 0.001 compared to T+EEQ 1; ddd, *p* < 0.001 compared to T+EDP 1. Scale bar = 100 µM.

Firstly, the effects of *t*-TUCB alone or with either 17,18-EEQ or 19,20-EDP on brown adipocyte differentiation were investigated. Murine brown preadipocytes were differentiated in the presence of the DMSO (the vehicle control), *t*-TUCB alone (T, 1 µM), or with 17,18-EEQ or 19,20-EDP at 1 or 10 µM for 6 days, as indicated in the Section 2 (during differentiation program). Lipid accumulation was analyzed by ORO-stained cell morphology, ORO absorbance, and brown marker gene protein expression. When used alone at 1 µM, *t*-TUCB showed minimal effects on ORO-stained brown adipocyte morphology, ORO absorbance, and brown marker protein expression compared to the (-) or the DMSO controls (Figure 1A–C). When with *t*-TUCB, 17,18-EEQ and 19,20-EDP in a dose-dependent manner increased the numbers of ORO-stained brown adipocytes and ORO absorbance, reaching statistical significance at 10 µM compared to *t*-TUCB alone and DMSO controls (Figure 1A,B). Moreover, with *t*-TUCB, 19,20-EDP at 10 µM significantly increased PGC1α protein expression, whereas 17,18-EEQ at 10 μM also significantly increased CD36 and UCP1 protein expression, in addition to PGC1α (Figure 1C). In contrast, 17,18-EEQ or 19,20-EDP alone had minimal effects on murine brown adipocyte differentiation (Figure S1).

### 3.2. 17,18-EEQ and 19,20-EDP Combined with t-TUCB Increase Mitochondrial Respiration and Proton Leak in Differentiating Murine Brown Adipocytes

**Figure 2:**
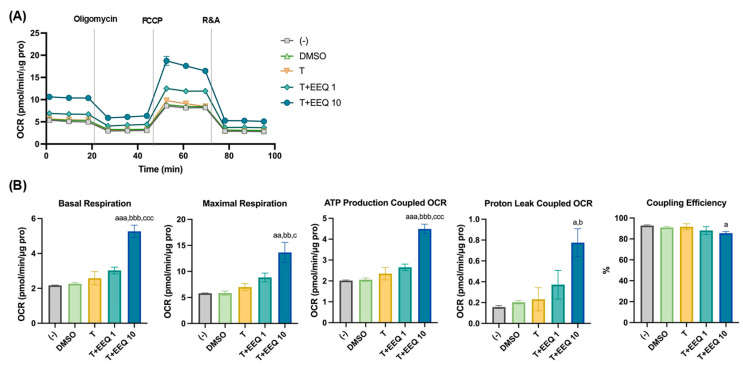
When combined with *t*-TUCB, 17,18-EEQ in a dose-dependent manner increased mitochondrial respiration and proton-leak coupled OCRs in differentiating murine brown adipocytes. Murine brown preadipocytes were differentiated in the presence of DMSO, *t*-TUCB alone (T), or 17,18-EEQ (T+EEQ) at 1 or 10 µM, as indicated for 4 days. Then, the cells were reseeded onto a 24-well XFe assay plate at 2.0 × 10^4^ cells per well. After 24 h, the cells were subjected to real-time OCR measurements. OCRs during mitochondrial stress tests (**A**) and bar graphs of basal respiration, maximal respiration, ATP production- and proton leak-coupled OCRs, and coupling efficiency (%) (**B**) are shown. Data = Mean ± SEM (n = 3–4). One-way ANOVA was used in (**B**). a, *p* < 0.05, aa, *p* < 0.01, aaa, *p* < 0.001 compared to DMSO; b, *p* < 0.05, bb, *p* < 0.01, bbb, *p* < 0.001 compared to T; c, *p* < 0.05, ccc, *p* < 0.001 compared to T+EEQ 1.

**Figure 3:**
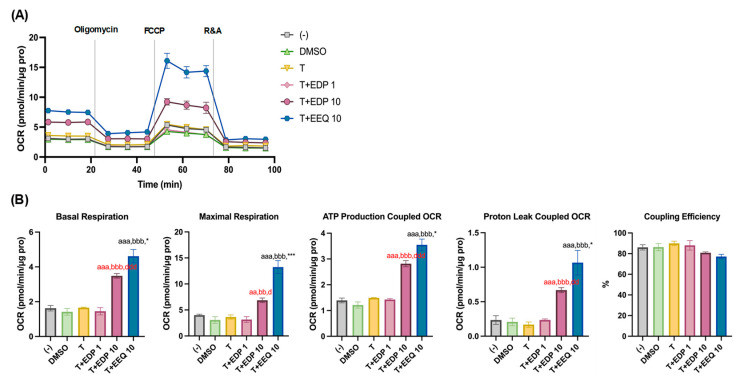
When combined with *t*-TUCB, 19, 20-EDP in a dose-dependent manner increased mitochondrial respiration and proton-leak coupled OCRs in differentiating murine brown adipocytes but is less potent compared to 17,18-EEQ. Murine brown preadipocytes were differentiated in the presence of DMSO, *t*-TUCB alone (T), or 19,20-EDP at 1 or 10 µM, as indicated for 4 days. Then, the cells were reseeded onto a 24-well XFe assay plate at 2.0 × 10^4^ cells per well. After 24 h, the cells were subjected to real-time OCR measurements. OCRs during mitochondrial stress tests (**A**) and bar graphs of basal respiration, maximal respiration, ATP production- and proton leak-coupled OCRs, and coupling efficiency (%) (**B**) are shown. Data = Mean ± SEM (n = 3–4). One-way ANOVA was used to compare among (-), DMSO, T, T+EDP 1, and T+EDP 10 in (**B**). Unpaired *t*-tests were used to compare T+EDP 10 and T+EEQ 10 in (**B**). aa, *p* < 0.01, aaa, *p* < 0.001 compared to DMSO; bb, *p* < 0.01, bbb, *p* < 0.001 compared to T; d, *p* < 0.05, dd, *p* < 0.01, ddd, *p* < 0.001 compared to T+EDP 1; *, *p* < 0.05, ***, *p* < 0.001, compared to T+EDP 10.

To confirm whether the effects of 17,18-EEQ or 19,20-EDP with *t*-TUCB on brown adipocyte differentiation are associated with increased thermogenic function, mitochondrial stress tests were performed by measuring cellular energetics using an XFe24 Extracellular Flux Analyzer (Figure 2 and Figure 3). Due to the limited number of wells per XFe24 plate, we analyzed dose responses of 17,18-EEQ and 19,20-EDP on separate plates (Figure 2 and Figure 3) and kept the higher dose of both epoxides (10 µM) on the same plate (Figure 3). Consistently, when used alone at 1 µM, *t*-TUCB had minimal effects on mitochondrial basal or maximal respiration, OCRs coupled with ATP production or proton leak, and coupling efficiency (Figure 2 and Figure 3). With *t*-TUCB, 17,18-EEQ-treated brown adipocytes during differentiation showed similar dose-dependent increases in basal and maximal mitochondrial respiration and OCRs coupled with ATP production and proton leak (Figure 2A,B), reaching statistical significance at 10 µM of 17,18-EEQ compared to *t*-TUCB alone or DMSO. 17,18-EEQ at 10 µM also showed a significant decrease in coupling efficiency compared to DMSO (Figure 2B).

Similarly, with *t*-TUCB, 19,20-EDP-treated brown adipocytes during differentiation showed significant dose-dependent increases in basal and maximal mitochondrial respiration and OCRs coupled with ATP production and proton leak, reaching statistical significance at 10 µM of 19,20-EDP, compared to *t*-TUCB alone or DMSO (Figure 3A,B).

However, between the two *n*-3 EpFAs, 17,18-EEQ showed significantly more potent effects on basal and maximal respiration and OCRs coupled to ATP production and proton leak than 19,20-EDP at the same dose of 10 µM (with *t*-TUCB) (Figure 3A,B). No significant differences were detected in coupling efficiency between the two EpFAs (Figure 3A,B).

Together, these results demonstrate that *t*-TUCB, 17,18-EEQ, and 19,20-EDP effectively promote murine brown adipogenesis, accompanied by increased mitochondrial respirations and uncoupling. Moreover, 17,18-EEQ is more potent than 19,20-EDP in promoting brown adipogenesis with increased thermogenic activities.

### 3.3. 17,18-EEQ with t-TUCB Upregulates Thermogenic and Lipid Metabolic Genes and Promotes Mitochondrial Respiration and Uncoupling in Differentiated Murine Brown Adipocytes

**Figure 4:**
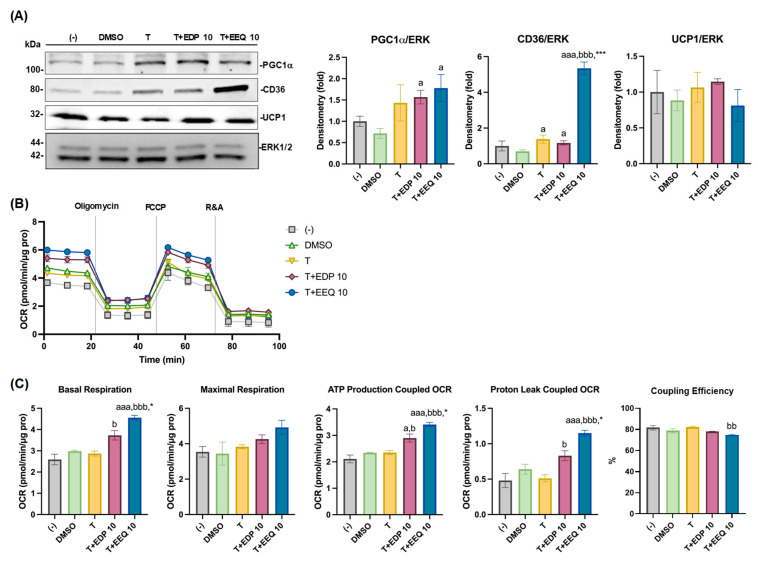
Combined with *t*-TUCB, 17,18-EEQ and 19,20-EDP differentially regulated protein expression and thermogenic function in differentiated brown adipocytes. Murine brown preadipocytes were differentiated for 6 days and then were treated with DMSO, *t*-TUCB alone (T), or 17,18-EEQ (T+EEQ) or 19,20-EDP (T+EDP) for 3 days. The protein expression of PGC1α, CD36, and UCP1 and the loading control ERK1/2 is shown in (**A**). Quantification of each protein expression by densitometry is shown on the right in (**A**). OCRs during mitochondrial stress tests were shown in (**B**), and bar graphs of basal respiration, maximal respiration, ATP production- and proton leak-coupled OCRs, and coupling efficiency (%) are shown in (**C**). Data = Mean ± SEM (n = 3–4). Unpaired *t*-tests were used in (**A**). One-way ANOVA was used in (**C**). a, *p* < 0.05, aaa, *p* < 0.001 compared to DMSO; b, *p* < 0.05, bb, *p* < 0.01, bbb, *p* < 0.001 compared to T; *, *p* < 0.05, ***, *p* < 0.001 compared to T+EDP 10.

The effects of 17,18-EEQ and 19,20-EDP combined with *t*-TUCB on differentiated brown adipocyte thermogenic function were further investigated. We treated differentiated murine brown adipocytes with the DMSO, *t*-TUCB alone (T), or 10 µM of 17,18-EEQ (T+EEQ) or 19,20-EDP (T+EDP) for 3 days, as described in the Section 2 (the post differentiation program). Brown marker gene protein expression was analyzed, and mitochondrial stress tests were performed (Figure 4A–C). PGC-1α protein expression was significantly increased by either EpFA with *t*-TUCB, but not by *t*-TUCB alone, compared to DMSO (Figure 4A). CD36 protein expression was significantly increased by *t*-TUCB alone or either epoxide with *t*-TUCB; however, 17,18-EEQ with *t*-TUCB showed much more robust effects than those for 19,20-EDP (with *t*-TUCB) and *t*-TUCB alone (Figure 4A). None of three treatments significantly affected UCP1 protein expression compared to DMSO (Figure 4A).

Consistently, *t*-TUCB alone showed minimal effects on mitochondrial respiration in the differentiated brown adipocytes (Figure 4B,C). With *t*-TUCB, both epoxides (at 10 µM) significantly increased basal, but not maximal, respiration and OCRs coupled with ATP production and proton leak in differentiated brown adipocytes compared to *t*-TUCB alone (Figure 4C); however, with *t*-TUCB, 17,18-EEQ had significantly more potent effects than 19,20-EDP (Figure 4C). Moreover, 17,18-EEQ, but not 19,20-EDP, significantly decreased coupling efficiency compared to *t*-TUCB alone in differentiated brown adipocytes (Figure 4C).

### 3.4. 17,18-EEQ and 19,20-EDP Activate PPARγ and Inhibit LPS-Induced NFκB Activation in Murine Brown Preadipocytes

**Figure 5:**
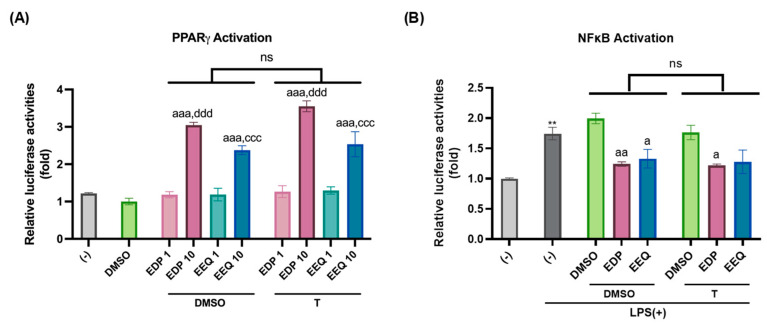
17,18-EEQ and 19,20-EDP activated PPARγ and inhibited LPS-induced NFκB activation in murine brown preadipocytes. Murine brown preadipocytes were seeded onto 24-well plates at 5.0 × 10^4^ cells per well. On the next day, cells were transiently transfected with murine PPARγ (**A**) or NFκB (**B**) transactivation reporters and β-gal plasmid for 24 h. The cells were treated with the DMSO, 17,18-EEQ (1 or 10 μM), or 19,20-EDP (1 or 10 μM) in the presence or absence of *t*-TUCB (1 μM) for 24 h in (**A**) or were pre-treated with DMSO. 17,18-EEQ (10 µM) or 19,20-EDP (10 µM) in the presence or absence of *t*-TUCB (1 μM) for 1 h, then co-treated with LPS for 18 h in (**B**). Reporter gene assays were performed and normalized to β-gal activities. Relative luciferase activities were expressed as folds of the (-) (set as 1). Data = Mean ± SEM (n = 3). Two-way ANOVA was used in (**A**,**B**). a, *p* < 0.05, aa, *p* < 0.01, aaa, *p* < 0.001 compared to DMSO, ccc, *p* < 0.001, compared to EEQ 1; ddd, *p* < 0.001 compared to EDP 1; **, *p* < 0.01 compared to (-). ns, not significantly different.

The molecular mechanisms by which 17,18-EEQ or 19,20-EDP with *t*-TUCB promote murine brown differentiation were then explored. Since PPARγ is one of the master regulators of brown adipocyte differentiation [25,26], the abilities of 17,18-EEQ and 19,20-EDP alone or with *t*-TUCB to activate PPARγ were investigated using murine PPARγ transactivation reporter assays (Figure 5A). Both 17,18-EEQ and 19,20-EDP alone significantly activated PPARγ at 10 µM, and the addition of *t*-TUCB did not significantly affect their effects (Figure 5A).

Since 17,18-EEQ or 19,20-EDP combined with *t*-TUCB delivered via osmotic pumps also showed the suppression of NFκB activation induced by high-fat diet-induced obesity in the iBAT [10], the effects of either EpFA alone or with *t*-TUCB were further investigated on lipopolysaccharide (LPS)-induced NFκB activation in brown preadipocytes (Figure 5B). LPS is the synthetic ligand for a prominent innate immune receptor toll-like receptor 4 (TLR4), found to be expressed and functional in murine brown preadipocytes [27]. At 10 μM, either 17,18-EEQ or 19,20-EDP alone significantly suppressed the LPS-induced NFκB activation (Figure 5B). Similarly, the addition of *t*-TUCB did not enhance anti-NFκB activities of either epoxide (Figure 5B).

Together, these results demonstrate that 17,18-EEQ and 19,20-EDP activate PPARγ and suppress NFκB, independent of the addition of the sEH inhibitor *t*-TUCB.

### 3.5. The Effects of PPARγ Knockdown on the Brown Adipocyte Differentiation Treated by 17,18-EEQ and 19,20-EDP Combined with t-TUCB

**Figure 6:**
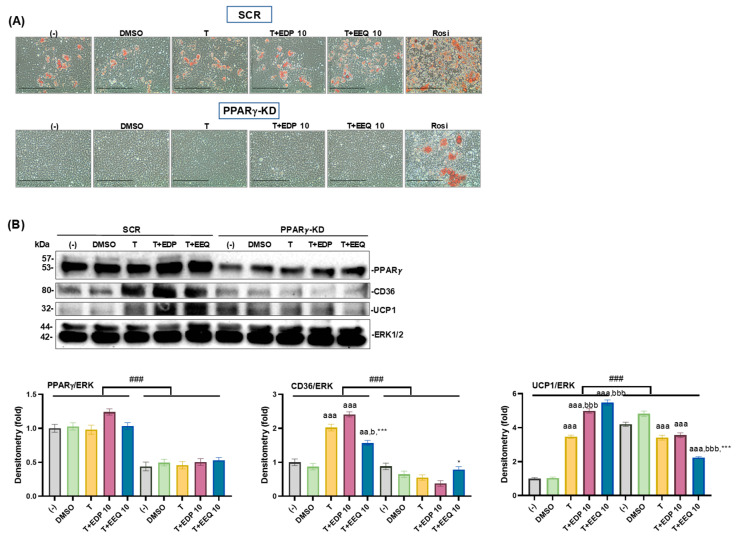
The effects of PPARγ knockdown on the brown adipocyte differentiation treated by *t*-TUCB alone or combined with 17,18-EEQ and 19,20-EDP. PPARγ-KD and SCR cells were differentiated in the presence or absence of DMSO, *t*-TUCB alone (T), and with 17,18-EEQ (T+EEQ) or 19,20-EDP (T+EDP). ORO-stained brown adipocyte morphology is shown in (**A**). Protein expression of brown adipocyte markers PPARγ, CD36, and UCP1 and the loading control ERK1/2 are shown in (**B**). Quantification of each protein expression by densitometry is shown below. Data = Mean ± SEM (n = 3 of technical replicates). One-way ANOVA was used to analyze the effects of T alone or combined with epoxides within each cell type. Two-way ANOVA was used to analyze the knockdown effects. aa, *p* < 0.01, aaa, *p* < 0.001 compared to DMSO; b, *p* < 0.05, bbb, *p* < 0.001 compared to T; *, ***, *p* < 0.05 and *p* < 0.001 compared to T+EDP 10; ###, significant differences between the SCR and PPARγ-KD cells. Scale bar = 100 µM.

To explore the roles of PPARγ in the beneficial effects of 17,18-EEQ and 19,20-EDP combined with *t*-TUCB on brown adipocyte differentiation, we employed murine brown preadipocyte cell line with PPARγ knockdown (KD) and their scrambled controls (SCR). PPARγ knockdown decreased basal differentiation, as shown by the decreased numbers of ORO-stained brown adipocytes in all groups compared to the SCRs (Figure 6A). PPARγ protein expression was significantly attenuated in the PPARγ-KD cells (Figure 6B) and so were the responses to rosiglitazone (Rosi, a potent PPARγ agonist) (Figure 6A and Figure S2A), both of which confirmed the knockdown in the PPARγ-KD cells.

Similarly, PPARγ knockdown significantly attenuated the effects of T alone or combined with 17,18-EEQ or 19,20-EDP on CD36 and UCP1 protein expression (Figure 6B). In contrast, PGC1α protein expression was not affected by T alone or combined with 17,18-EEQ or 19,20-EDP in the SCR cells, nor in the PPARγ-KD cells (Figure S2B).

### 3.6. The Effects of PPARγ Antagonism on the Differentiated Brown Adipocytes Treated with 17,18-EEQ and 19,20-EDP Combined with t-TUCB

**Figure 7:**
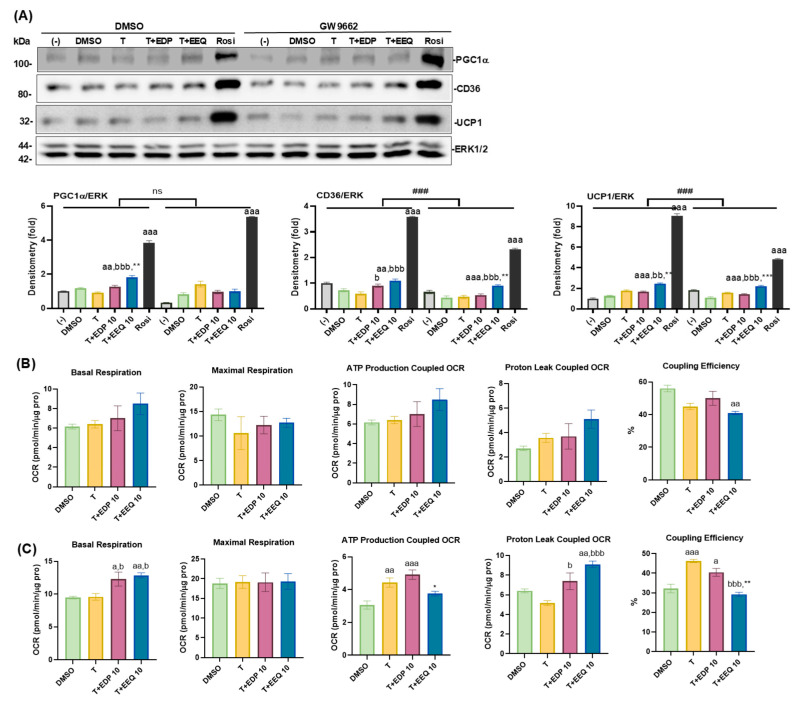
The effects of PPARγ antagonist GW9662 on protein expression and thermogenic function induced by t-TUCB alone or combined with epoxides in differentiated brown adipocytes. Murine brown preadipocytes were differentiated, pre-treated with GW9662 or DMSO, and then co-treated with DMSO, *t*-TUCB alone (T), or with 17,18-EEQ (T+EEQ) or 19,20-EDP (T+EDP) for 3 days. Protein expression of PGC1α, CD36, and UCP1 and the loading control ERK1/2 are shown in (**A**). Quantification of each protein expression by densitometry is shown below, and Data = Mean ± SEM (n = 3 of technical replicates) (**A**). Bar graphs of basal respiration, maximal respiration, ATP production- and proton leak-coupled OCRs, and coupling efficiency (%) of the DMSO- or GW9662-treated cells are shown, and Data = Mean ± SEM (n = 3–6) in (**B**,**C**), respectively. One-way and two-way ANOVA were used in (**A**) and one-way ANOVA was used in (**B**,**C**). a, *p* < 0.05, aa, *p* < 0.01, aaa, *p* < 0.001 compared to DMSO; b, *p* < 0.05, bb, *p* < 0.01, bbb, *p* < 0.001 compared to T; **, *p* < 0.01, ***, *p* < 0.001 compared to T+EDP 10. ###, significant differences between the DMSO- and GW9662-treated cells.

To further explore the PPARγ antagonism in the beneficial effects on the differentiated brown adipocytes due to 17,18-EEQ and 19,20-EDP combined with *t*-TUCB, we used a common and potent PPARγ antagonist GW9662 [28]. We found that GW9662 at 20 µM almost completely suppressed the effects of rosiglitazone (Rosi, a potent PPARγ agonist) in promoting brown adipocyte differentiation, compared to BADGE, another PPARγ antagonist (Figure S3). GW9662 significantly suppressed Rosi-induced increases in the protein expression of CD36 and UCP1, but not PGC1α, in the differentiated brown adipocytes, but had modest effects on CD36 and UCP1 protein expression increased by 17,18-EEQ or 19,20-EDP combined with *t*-TUCB (Figure 7A). In contrast, the PGC1α protein expression increased by T+EEQ was abolished by GW9662 (Figure 7A). Moreover, GW9662 significantly increased OCRs coupled with ATP production and coupling efficiencies in T- and T+EDP-treated groups, but had minimal effects on basal respiration and OCRs coupled with proton leaks in T+EDP and T+EEQ groups (Figure 7B,C).

## 4. Discussion

In the current studies, 17,18-EEQ and 19,20-EDP, when stabilized by *t*-TUCB, were demonstrated to significantly promote murine brown adipocyte differentiation, accompanied by increased mitochondrial respiration and uncoupling. Moreover, both EpFAs with *t*-TUCB significantly promote mitochondrial respiration and uncoupling (i.e., thermogenesis) of differentiated brown adipocytes. In both cell types, 17,18-EEQ shows more potent effects than 19,20-EDP, supported by the increased protein expression of thermogenic genes (PGC1α and UCP1) and/or the fatty acid transporter CD36. Furthermore, 17,18-EEQ and 19,20-EDP were shown to activate PPARγ and suppress LPS-induced NFκB activation, regardless of *t*-TUCB’s presence, in murine brown preadipocytes.

To our knowledge, this is the first report demonstrating the promotion of brown adipogenesis by 17,18-EEQ and 19,20-EDP with *t*-TUCB with increased mitochondrial respiration and uncoupling. 17,18-EEQ and 19,20-EDP are major EpFAs from EPA and DHA, respectively. Supplements of EPA and DHA in the forms of fatty fish or fish oil with different formulations or enrichments have been reported to increase thermogenesis and heat production, leading to the protection against diet-induced obesity and associated metabolic dysfunction in animals [13,14]. In addition, *n*-3 PUFA, especially EPA, has been shown to promote cellular brown adipogenesis [16,17] and browning in vitro [15,29]. Therefore, our results are consistent with previous reports and further suggest that 17,18-EEQ and 19,20-EDP may be effective metabolites responsible for EPA- or fish oil-induced increases in energy expenditure and thermogenesis.

Fish oil, typically a mixture of EPA and DHA, has been reported to beneficially modulate gene expression in fat tissue and promote energy expenditure and thermogenesis in animals [30,31,32]. However, whether the beneficial effects of fish oil come from EPA, DHA, or both are largely uncharacterized. In addition, few studies directly compared the effects between EPA and DHA. One study reported that EPA increased thermogenic gene expression, mitochondria function, and β-oxidation in murine brown adipocytes and promoted the browning process in inguinal white adipocytes, whereas no effects on thermogenic gene expression were observed in DHA-treated murine brown and inguinal white adipocytes [15]. In the current study, with *t*-TUCB, both 19,20-EDP and 17,18-EEQ promoted brown adipocyte differentiation with increased mitochondrial respiration and uncoupling; however, 17,18-EEQ showed more potent effects than 19,20-EDP in that 17,18-EEQ additionally increased UCP1 and CD36 protein expression and almost doubled proton leak-coupled OCR in the treated brown adipocytes compared to 19,20-EDP. Moreover, with *t*-TUCB, 17,18-EEQ increased proton leak-coupled OCRs and coupling efficiency and increased CD36 protein expression in differentiated brown adipocytes compared with 19,20-EDP. These in vitro results align with our previous study, which demonstrated the potentials of 17,18-EEQ or 19,20-EDP with *t*-TUCB in increasing core body temperature and improving cold tolerance in diet-induced obese mice [10].

How *n*-3 EpFAs activate brown adipogenesis and increase thermogenesis needs further studies. Studies have suggested that EPA may act through membrane-bound G-protein-coupled receptor 120 (GPR120), Fibroblast Growth Factor 21 (FGF21) production, and mitochondrial biogenesis regulatory pathways such as AMPK-SIRT-PGC1 [14]. Some biological effects of PUFAs were thought to be mediated through PPARs, including PPARγ, in various disease models [33]. For example, 17,18-EEQ was reported to suppress inflammation in the bronchi to protect lung function in a PPARγ-dependent manner [34]. PPARγ is one of the master regulators of brown adipogenesis and thermogenesis [25,26]. However, no studies have reported the effects of *n*-3 EpFAs on PPARγ activation in brown preadipocytes. The results herein showed that both 17,18-EEQ and 19,20-EDP significantly activated PPARγ reporter in brown preadipocytes, suggesting that 17,18-EEQ and 19,20-EDP may promote brown adipocyte differentiation through, at least in part, PPARγ activation.

We further studied the roles of PPARγ underlying the epoxides’ effects. PPARγ knockdown significantly decreased PPARγ protein expression and attenuated the cells’ responses to Rosi. The effects of epoxides with *t*-TUCB on brown differentiation were attenuated by PPAR knockdown, as indicated by the decreased numbers of ORO-stained brown adipocytes and attenuated CD36 and UCP1 protein expression in PPARγ-KD cells. On the other hand, PPARγ antagonism by GW9662 had modest to minimal effects on the impacts of epoxides with *t*-TUCB on CD36 and UCP1 protein expression and thermogenic function in the differentiated brown adipocytes. It is noted that there were differences in response to the epoxides with *t*-TUCB in the PPARγ knockdown (Figure 6) and GW9662 (Figure 7) experiments, compared to the parental cells (Figure 1 and Figure 4). The differences could be due to the impact of cell manipulation during the generation of the knockdown and additional DMSO added as the vehicle control for GW9662. Future studies are needed to confirm the roles of PPARγ in mediating epoxides’ effects on brown differentiation and characterize the mechanisms by which epoxides’ impact on the differentiated brown adipocytes.

On the other hand, the activation of innate immune receptors, such as TLR4 or nucleotide-binding oligomerization domain-containing protein 1 (NOD1), or pro-inflammatory cytokines, such as tumor necrosis factor α (TNFα), were reported to impair brown adipocyte differentiation [27,35]. NFκB is one of the major transcription activators mediating inflammatory responses in brown adipocytes [36]. Using the NFκB reporter, we demonstrated that 17,18-EEQ or 19,20-EDP significantly suppressed NFκB activation in brown preadipocytes, suggesting that 17,18-EEQ or 19,20-EDP may contribute to their beneficial effects of promoting brown adipocyte differentiation via suppressing NFκB. However, the detailed molecular mechanisms by which *n*-3 epoxides intercept the interactions between the NFκB pathway and brown adipocyte differentiation need further investigation.

It is worth noting that the 17,18-EEQ and 19,20-EDP’s abilities to activate PPARγ and suppress NFκB are independent of *t*-TUCB’s presence. Our results that 17,18-EEQ or 19,20-EDP with *t*-TUCB, but not alone (Figure S1), promote brown adipogenesis are consistent with previous reports that show the potentiation of *n*-3 epoxides’ effects by the sEH inhibitor [8,11,12].

In addition to brown adipogenesis, we demonstrate the effects of 17,18-EEQ and 19,20-EDP with *t*-TUCB on differentiated brown adipocytes. Compared to *t*-TUCB, which showed minimal effects on mitochondrial respiration, both epoxides with *t*-TUCB significantly increased basal, but not maximal, respiration, and OCRs coupled with ATP production and proton leak in differentiated brown adipocytes. 17,18-EEQ (with *t*-TUCB) showed more potent effects than 19,20-EDP on basal respiration, OCRs from proton leak, and coupling efficiency in differentiated brown adipocytes. The facts that PPARγ antagonism by GW9662 had minimal impact on the epoxides’ effects suggest that other mediators/pathways might be involved. For example, 17,18-EEQ (with *t*-TUCB) induced higher CD36 protein expression in differentiated brown adipocytes. CD36 is a fatty acid transporter on the cell surface, responsible for fatty acid uptake from circulation. CD36 expression was upregulated in the BAT by the cold exposure, contributing to cold-induced triglyceride clearance [37]. Conversely, CD36^−/−^ mice showed impaired cold tolerance with decreased fatty acid uptake and thermogenic gene expression in the BAT after cold exposure [38]. Therefore, a more potent induction of CD36 protein expression by 17,18-EEQ (with *t*-TUCB) may explain its more potent effects on mitochondrial uncoupling than 19,20-EDP in differentiated brown adipocytes. The roles of CD36 in mediating the epoxides’ effects in differentiated brown adipocytes need further investigation.

## 5. Conclusions

The current study demonstrates that, with the sEH inhibitor *t*-TUCB, 17,18-EEQ and 19,20-EDP promote brown adipogenesis and thermogenic function in vitro, and 17,18-EEQ may be more potent than 19,20-EDP in promoting brown adipogenesis and brown adipocyte thermogenic function. Moreover, 17,18-EEQ and 19,20-EDP activate PPARγ and suppress NFκB activation. Furthermore, PPARγ may be required for the effects of epoxides on differentiation but not on the thermogenic function post differentiation. Translational studies of these *n*-3 epoxides on human brown adipocyte differentiation and functions are warranted.

## Data Availability

The original contributions presented in the study are included in the article/Supplementary Material, further inquiries can be directed to the corresponding author.

## Supplementary Materials

The following supporting information can be downloaded at: https://www.mdpi.com/article/10.3390/nu17060936/s1. Figure S1: 17,18-EEQ and 19,20-EDP alone had minimal effects on murine brown adipocyte differentiation; Figure S2: The effects of PPARγ knockdown on murine brown adipocyte differentiation in the presence or absence of Rosi, *t*-TUCB alone or combined with epoxides; Figure S3: PPARγ antagonist GW9662 suppressed rosiglitazone (Rosi)’s effects on murine brown adipocyte differentiation.
