# Supplementary material for: Effects of 17,18-Epoxyeicosatetraenoic Acid and 19,20-Epoxydocosapentaenoic Acid Combined with Soluble Epoxide Hydrolase Inhibitor t-TUCB on Brown Adipogenesis and Mitochondrial Respiration
Source: Nutrients. 2025 Mar 7;17(6):936. doi: 10.3390/nu17060936 (PMC11946110; doi:10.3390/nu17060936)
Supplement: Supplementary file 1 [file nutrients-17-00936-s001.zip › nutrients-3425312-supplementary.pdf]

nutrients

Article

Effects of 17,18-Epoxyeicosatetraenoic Acid and Epoxydocosapentaenoic Acid Combined with Soluble Epoxide Hydrolase Inhibitor *t*-TUCB on Brown Adipogenesis and Mitochondrial Respiration

Yang Yang [1,#] Haoying Wu [1], Xinyun Xu [1], Christophe Morisseau [2], Kin Sing Stephen Lee [3], Bruce D. Hammock [2], Jiangang Chen [4], and Ling Zhao [1,*]

Supplemental Materials

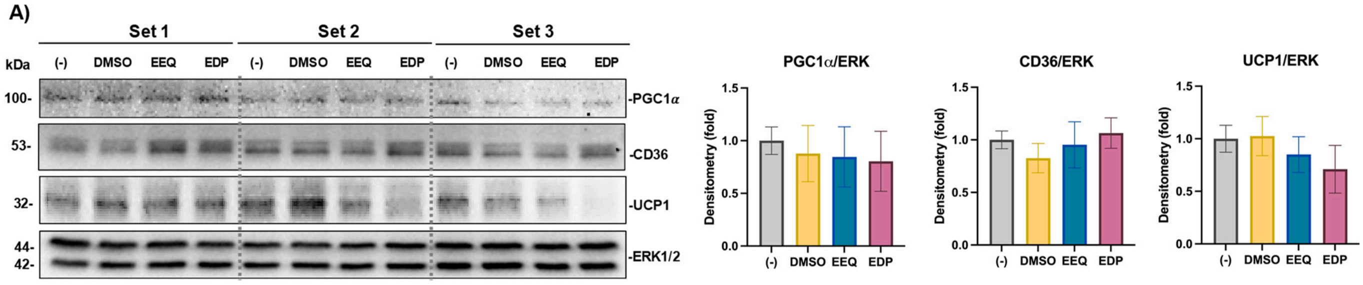

Figure S1. 17,18-EEQ and 19,20-EDP alone had minimal effects on murine brown adipocyte differentiation. Murine brown preadipocytes were differentiated in the presence or absence of DMSO (the vehicle control), 17,18-EEQ (10 μM) or 19,20-EDP (10 μM) for 6 days. Protein expression of brown adipocyte marker genes, PGC1α, CD36, UCP1, and the loading control ERK1/2 were shown on the left. Quantification of each protein expression by densitometry is shown on the right. One-way ANOVA was used. No significant differences were detected.

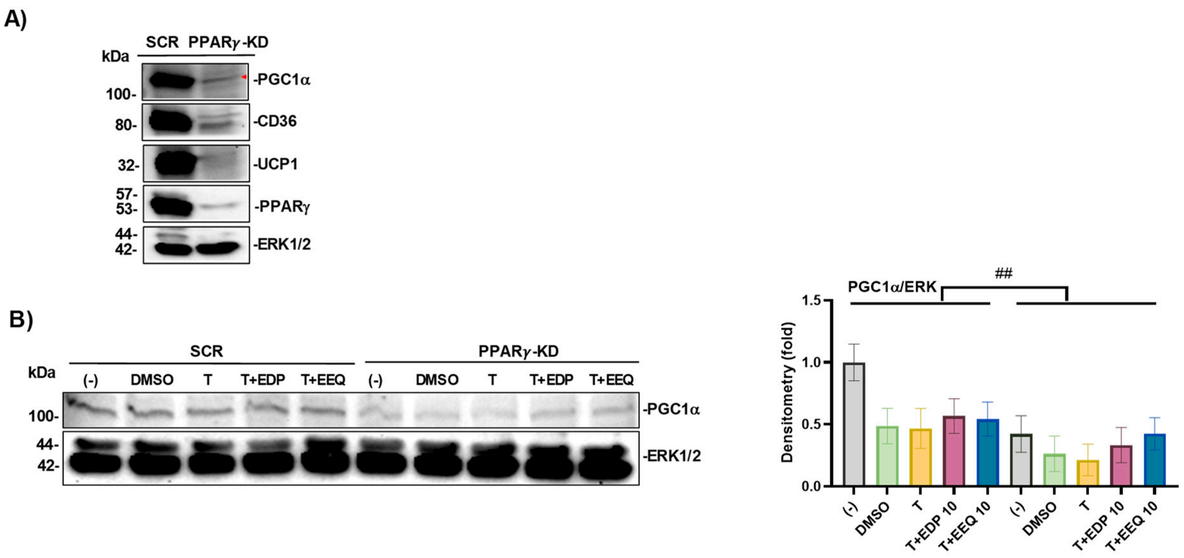

Figure S2. The effects of PPARγ knockdown on murine brown adipocyte differentiation in the presence or absence of Rosi, t-TUCB alone or combined with epoxides. PPARγ-KD and SCR cells were differentiated in the presence or absence of Rosi, DMSO, *t*-TUCB alone (T), or with 17,18-EEQ (T+EEQ) or 19,20-EDP (T+EDP). The effects of Rosi (A), T alone or combined with epoxides (B) on brown adipocyte differentiation were compared in the PPARγ-KD and their SCR controls. Protein expression of various brown markers and the loading control ERK1/2 are shown. Quantification of PGC1α protein expression by densitometry is shown for (B). Data=Mean±SEM (n=3 of technical replicates). One-way ANOVA was used to analyze the effects of T alone or combined with the epoxides within each cell type. Two-way ANOVA was used to analyze the knockdown effects. No significant differences were detected for T alone or combined with epoxides; ##, significant differences between the SCR and PPARγ-KD cells with $p<0.01$.

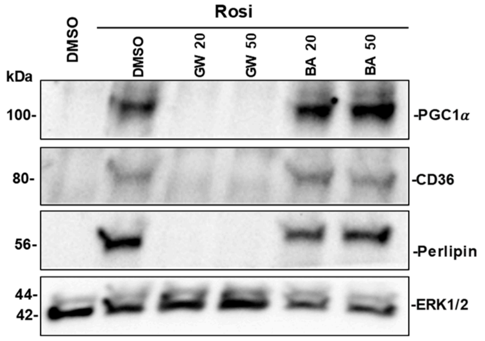

Figure S3. PPARγ antagonist GW9662 suppressed rosiglitazone (Rosi)'s effects on murine brown adipocyte differentiation. Murine brown preadipocytes were differentiated in the differentiation media supplemented with Rosi (1 μM) in the presence of GW9662 (GW) (20, 50 μM), BADGE (BA) (20, 50 μM). Protein expression of brown marker genes PGC1α, CD36, and perilipin and the loading control ERK1 and 2 were shown.
